# Electrical injury and the long‐term risk of cataract: A prospective matched cohort study

**DOI:** 10.1111/aos.15220

**Published:** 2022-07-27

**Authors:** Anette Kærgaard, Kent J. Nielsen, Ole Carstensen, Karin Biering

**Affiliations:** ^1^ Department of Clinical Medicine Aarhus University Aarhus Denmark; ^2^ Department of Occupational Medicine – University Research Clinic Danish Ramazzini Centre Goedstrup Hospital Herning Denmark

**Keywords:** cataract, cohort study, electric cataract, electric shock, electrical injuries

## Abstract

**Purpose:**

Over the years, many cases of electric cataract related to severe electrical injuries have been reported. Most have been cases where the entrance or exit point of the current was on the skull or near the eyes. Still, cases of cataract have been reported where an electric current has passed through the body between two contact points remote from the eyes. This study investigates whether persons exposed to an electric current develop cataracts in the subsequent years.

**Methods:**

We identified 14 112 persons who had received electrical injuries in two Danish registries. We matched these with patients partly with dislocation/sprain injuries and partly with persons from the workforce from the same occupation using year of accident, sex and age as matching variables in a prospective, matched‐cohort design. We identified cataract as outcome (DH25, DH26 and DH28) in the Danish National Patient Registry. The associations were analysed using conditional Cox and logistic regression.

**Results:**

We did not identify an increased risk of cataract following electrical injury compared to matched controls.

**Conclusion:**

A review of the literature clearly substantiates the occurrence of electric cataract as a consequence of electric current coming in contact with a point on the skull or near the eye. However, our results indicate that electric cataract is not a delayed‐onset effect of electrical injury, in general, and do not suggest a need for cataract screening in all cases of electrical injury.

## 1 INTRODUCTION

The consequences of electrical injuries are complex. Immediate, familiar clinical consequences are burns, deep tissue damage and cardiac effects. In particular, burn and trauma centres report clinical data on these patients. Cataract is another frequently described complication of electrical injury. The pathology and clinical characteristics of numerous cases of electric cataract have been described in detail in the ophthalmology literature for over a century (Boozalis et al., 1991; Franklin & Cordes, 1925; Gjessing, 1922; Hanna & Fraunfelder, 1972; Hashemi et al., 2008).

An electric shock is experienced when an electric current passes through a part of the body from an entry point to an exit point. Interacting variables include voltage, amperage, tissue resistance, type of current, current pathway, site and duration of contact, which determine the type and severity of the injuries (Duff & McCaffrey, 2001). Usually, only the voltage is known and reported. Amperage, which affects tissue damage and mortality, is usually undetermined because various resistances in the circuit are unknown.

In 1962, J.C. Long reviewed 63 published cases of electric cataract. In most cases, the entry point was on the skull or near the eyes, and the voltage varied from 220 to 60 000 volts (Long, 1962).

A visual demonstration of ocular lens changes due to electricity was initiated in 1972 by Fraunfelder and Hanna, who published the first serial slit‐lamp photographs of electric cataracts, from the earliest lens changes to the development of mature, dense opacities (Fraunfelder & Hanna, 1972). In 2007 Grewal et al. used Scheimpflug imaging to document the earliest subclinical manifestations of an electric cataract (Grewal et al., 2007). The patient was a young electrician, and an electric wire (11 000 volts) had touched his scalp.

Saffle et al.'s review of the incidence and characteristics of cataracts in 113 patients who had suffered severe electrical injuries is frequently cited, and the 6.2% incidence of cataracts in this group has been referred to in contexts (Saffle et al., 1985).

In contrast, a 2017 clinical update concerning principal types of organ‐specific injuries following an electric shock did not even mention electric cataract (Waldmann et al., 2017).

In 2019, the first case of bilateral cataract was reported, where the onset, type and progression of the patient's cataracts were explained by an electrical cardioversion 2 weeks previously (Maghera et al., 2019). This finding complements earlier incidents that involved an electric shock with no contact points on the head, but instead a flow of electric current through the body between two contact points (hand–hand or hand–foot) (Biro & Pamer, 1994; Miller et al., 2002) or finger to shoulder (Martinez & Nguyen, 2000), and bilateral cataracts.

A review of the literature raises a clinically important question: Is screening for cataract as a potential complication of electrical injuries relevant in all cases of electrical injury, and not only in cases of high‐voltage injury and/or when the electric shock involves contact points near the eyes?

In our clinical practice in occupational medicine, we meet patients with sequelae to electrical injuries. These patients have not suffered severe acute trauma, but over time their accident has led to pain, peripheral nerve symptoms and/or memory and concentration impairment. For many years, we have surveyed patients for symptoms of cataract, but never met any patient with this complication, neither acute cases nor late onset.

This longstanding clinical observation, together with studies that reported cataracts that developed despite a lack of electrical contact with the head or eyes (Biro & Pamer, 1994; Martinez & Nguyen, 2000; Miller et al., 2002), suggested the need for this investigation.

This study investigated whether persons who sustain any form of electrical injury develop cataracts in subsequent years.

## 2 MATERIALS AND METHODS

This study was a matched cohort study based on electrical injuries registered in two population‐based registers: (1) the Danish National Patient Registry (DNPR), which covers all hospital contacts in Denmark, including information regarding diagnoses and procedures for both in‐ and outpatients and casualty department visits (Lynge et al., 2011; Schmidt et al., 2015); and (2) the Danish Working Environment Authority (DWEA), which maintains a registry of work injuries (2020).

The study covered registered Danish electrical injuries from 1994 to 2016. DNPR began to use ICD‐10 codes in 1994, and we had data up to 2016. DWEA covered work injuries from 2005 to 2017. We included electrical injuries from 1996 to 2014 to allow for at least 2 years of time clear of cataract and at least 2 years for cataracts to develop.

Injury records from DNPR and DWEA were linked to Statistics Denmark, the central authority for Danish registries and statistics, by a personal identification number (CPR number) and injury date/year. Each of the Danish citizen and registered migrant worker have a unique CPR number that links each person to demographic and work‐related registries (Schmidt et al., 2014).

### 2.1 Participants

In the DNPR, persons with an electrical injury, ICD‐10 diagnoses (DT754, EUHA10 and EUYZ203), were identified and both in‐ and outpatients were included.

In DWEA, persons with electrical injuries were identified by two different codes that define exposure: ‘acute/brief exposure to welding arc or electric arc’ and ‘acute/brief exposure to electricity or reception of electric charge in the body’.

If an accident was registered in both registries (±7 days), it was coded as one injury.

### 2.2 Other variables

Sex, age and occupation were derived from Statistics Denmark, as were dates of migration or death. We registered if the patient was part of the workforce at the time of injury. The injury, if identified in the DNPR, was not necessarily work‐related, whereas all injuries in DWEA were work‐related.

For all hospital admissions, we determined the total length of hospitalisation, including time in the casualty department. We used this as a proxy for injury severity, given the probability that the most severe accidents would result in the longest hospital stays. Not all injuries from DWEA could be assigned a length of hospitalisation as not all were hospitalised.

### 2.3 Matching

Each person with an electrical injury was matched in two different ways with persons from the same data source (DNPR or DWEA).

#### 2.3.1 Match 1: Injury match, dislocation/sprain

Each electrical injury patient was matched with up to 10 other patients with a dislocation/sprain (DS93 in the DNPR and ‘sprains’ in DWEA). The matching variables were sex, year of injury and age. For all matches, the match‐persons were randomly chosen, if more than 10 were available. The diagnosis of dislocation/sprain was chosen, since it is frequent and is not suspected to cause cataract.

#### 2.3.2 Match 2: Occupation match

Each electrical injury patient was matched with up to 10 other persons from the working population of the same occupational group, year, sex and age. The purpose of this match was to take into consideration whether persons in certain occupations were at higher risk of cataract due to socioeconomic factors or other occupational exposure than electric shocks. More detailed descriptions of the matching procedure were reported in an earlier publication (Biering et al., 2020).

### 2.4 Outcome

The outcome of cataract was defined by the ICD‐10 codes DH25, DH26 and DH28, including all subgroups. We excluded both electrical injury patients and match‐persons if they were registered with one of these diagnoses before the match‐day.

In the occupation match, all patients with an injury registered in DWEA were defined as part of the working population, as their injuries had occurred while working. However, not all were defined as part of the workforce in Statistics Denmark, probably because they had experienced the electrical injury while working a part‐time job (students, interns or retired persons). This meant that 93 persons with an injury registered in DWEA could not be matched in the occupation match.

Occupation and current work status were derived from the RAS register at Statistics Denmark using DISCO codes (Biering et al., 2020). DISCO is the official Danish version of the International Standard Classification of Occupations (ISCO) prepared by the International Labour Organization (ILO) (2020).

Both electrical injury patients and controls who emigrated or died during follow‐up were censored from that date.

### 2.5 Statistical methods

We compared the two matched groups using conditional logistic regression, where each matched group consisted of one injured person and up to 10 match‐persons, depending on availability and exclusion for previous cataract. We also conducted a conditional Cox regression, to examine the outcome in a time to event setting, to determine whether cataracts would occur earlier for patients who had experienced an electrical injury compared to controls.

The electrical injuries were a combination of occupational injuries and injuries in other settings. The DNPR did not provide information about the setting, but we tried to compensate for this with an additional analysis in the dataset matched on dislocation/sprain including only persons in the workforce at the time of the injury. In a sensitivity analysis, we also adjusted the analysis for length of hospitalisation in match 1.

### 2.6 Ethics

All procedures performed in this study were in accordance with Danish ethical standards and with the Declaration of Helsinki. The Regional Data Protection Agency has approved the study (reference number 1‐16‐02‐113‐18). According to the Danish law, register‐based studies only need approval from the ethics committee if the data include human biological material (§ 14 in ‘Promulgation of the Act on the ethical treatment of health science research projects and health data science research projects’ available in Danish from www.retsinformation.dk/eli/lta/2020/1338). Furthermore, patient consent in register studies is not needed.

## 3 RESULTS

**FIGURE 1:**
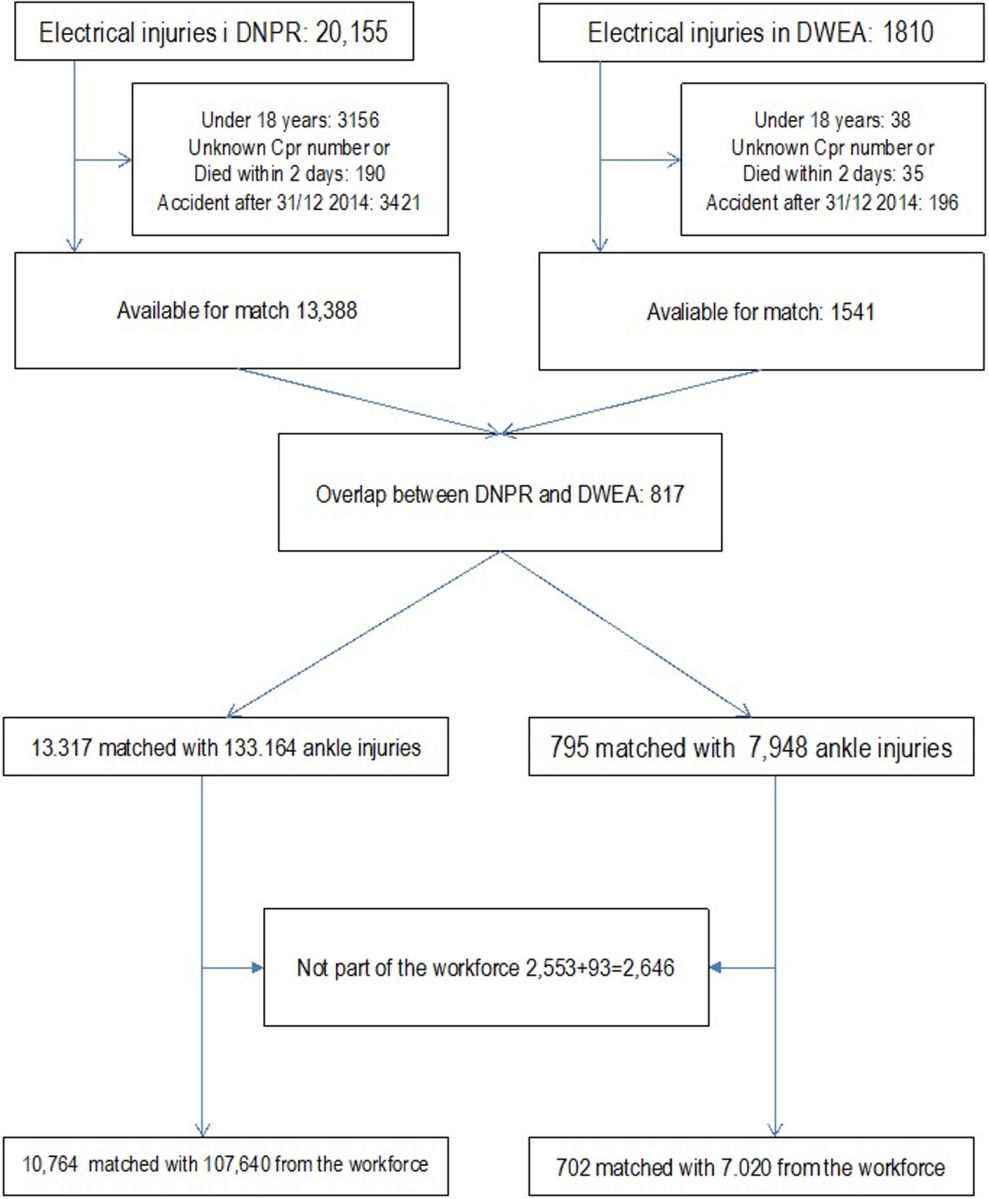
Descriptions of electrical injuries addressed in this study in the Danish National Patient Registry (DNPR) and the Danish Working Environment Authority (DWEA), exclusions and injury match.

We identified 20 155 electrical injuries in DNPR and 1810 in DWEA (Figure 1). After excluding persons under 18, persons without a valid CPR number, persons who died within the first 2 days following their accident and persons with accidents during the last 2 years of the study period, there was an overlap of 817 persons from the two registries. When we eliminated this overlap, we had 13 317 from DNPR and 795 from DWEA. For the occupation match, we excluded 2646 persons not in the workforce, resulting in 10 764 injuries from DNPR and 702 injuries from DWEA. A match with 10 match‐persons was possible for almost all electrical injuries (Figure 1).

The majority of the injuries happened to men, especially those registered with DWEA (Table 1), and to younger persons, which was most evident in DNPR (data not shown). The occupations held by those with the greatest number of electrical injuries were manual workers, but service workers/sales staff were also overrepresented, when compared to the distribution of occupations in Denmark (data not shown). In most cases, the length of hospitalisation was less than 2 days; however, a large proportion of electrical injuries registered in DWEA did not involve a hospital visit or involved only an outpatient visit (Table 1). Further details on the cohort characteristics were reported previously (Biering et al., 2020).

**TABLE 1:** Sex, hospitalisation, death rates and cataract diagnoses among the cohort with electrical injuries (*N* = 14 112)

	DNPR	DWEA^a^
*N*	*N* = 13 317	*N* = 795
Men (*n*/%)	10 180 (76.4%)	679 (85.4%)
Hospitalisation (*n*/%)		
Less than one day	9045 (67.9%)	193 (24.3%)
One day or more	2916 (21.9%)	154 (19.4%)
Missing/outpatient	1356 (10.2%)	448 (56.4%)
Died within 2 days of injury (*n*/%)	6 (0.1%)	<5 (<1%)
Cataract diagnosis before injury (*n*/%)	40 (0.4%)	<5 (<1%)
Cataract diagnosis 5 years after injury (*n*/%)	37 (1.0%)	6 (4.3%)
Cataract diagnosis full follow‐up^b^ (*n*/%)	122 (5.1%)	9 (8.3%)

Abbreviations: DNPR, The Danish National Patient Registry; DWEA, The Danish Working Environment Authority.

^a^ Overlap between registers removed.

^b^ Time from year of accident (between 1996 and 2014) to 2016.

The results of the regression analyses for both matches are presented in Table 2. We did not find an increased incidence of cataract following electrical injuries compared to controls. All estimates were highest in the occupation match, where the match persons were controls without injury, but had the same occupation. In the injury‐match with dislocation/sprain persons, we further restricted the analysis to persons from the workforce, and this increased the risk estimates OR:1.15[0.91;2.04]. The sensitivity analysis, where we adjusted for length of hospitalisation, gave a similar risk estimate HR:0.92[0.67;1.28].

**TABLE 2:** Associations between electrical injuries and cataract over the whole study and in intervals in two matched designs

Cataract	Time to event^c^	6 months^d^	12 months^d^	2 years^d^	3 years^d^	4 years^d^	5 years^d^
	HR	OR	OR	OR	OR	OR	OR
Match: Dislocation/sprain^a^	0.96 [0.79;1.16]	0.49 [0.12;2.04]	1.16 [0.62;2.18]	1.05 [0.65;1.70]	0.81 [0.53;1.25]	0.93 [0.66;1.33]	0.79 [0.57;1.11]
Match: Occupation^b^	1.23 [0.98;1.54]	1.15 [0.27;5.09]	2.12 [0.93;4.81]	1.53 [0.83;2.82]	1.13 [0.66;1.93]	1.27 [0.82;1.99]	1.06 [0.69;1.64]

Abbreviations: HR, hazard ratio; OR, odds ratio.

^a^ Each electrical injury patient was matched with up to 10 other patients with a dislocation/sprain on sex, year of injury and age.

^b^ Each electrical injury patient was matched with up to 10 other patients within the same occupation group on sex, year of injury and age.

^c^ From date of injury (1996–2014) to 2016.

^d^ From date of injury and x‐months/years forward.

## 4 DISCUSSION

To the best of our knowledge, this study is the first attempt to investigate the association between exposure to electrical injury, in general, and the development of cataracts. Previous studies comprise case reports and selected patient populations who were often admitted to burn centres, so we cannot compare our results directly to previous studies.

We investigated 14 112 diagnosed electrical injuries over a period of 19 years, and we found no increased risk of being diagnosed with cataracts in the subsequent years compared to matched controls. The lack of association was consistent, from within 6 months following the injury to throughout the entire study period, also when the analysis was restricted to a working population.

### 4.1 Limitations

Mandatory registration of accidents in DNPR began in 2000. Before that, the diagnosis code of DT754 was sometimes used, but not necessarily, if the main problem following the accident was something else, such as a burn or unconsciousness. This may have led to underreporting of electrical injuries, especially during the first years of this study. Even today, underreporting of diagnosis code DT754 may be a problem, as detailed registration in the casualty departments may be deprioritised in acute situations. If the code for the more serious electrical injuries is not reported in the DNPR, and instead burns or amputations (for example) are registered, the association may be underestimated. However, if an inadequate coding practice does leave us without the more serious electrical injuries, the remaining group actually includes the patients in which we are interested, as we wanted to investigate whether persons exposed to electrical injury in general are diagnosed with cataracts in the subsequent years. Based on numerous case reports (Boozalis et al., 1991; Franklin & Cordes, 1925; Gjessing, 1922; Hanna & Fraunfelder, 1972; Hashemi et al., 2008), we know that high‐voltage electrical injuries and/or electrical injuries with contact points on the skull or near the eye are evidently associated with the development of electric cataracts. The result of this study does not alter that.

In DWEA of accidents, too, underreporting is a well‐known problem (Lander et al., 2014). Thus, the true incidence of electrical injuries is impossible to determine, as the number of registered accidents reflects registration practices in both register settings. However, since this study was based on matching, underreporting in the two data sources has probably not biased the result. If the registered injuries differ in type, severity or duration from those not registered, it may cause bias in an unknown direction. Severity and other characteristics such as voltage are not registered in DNPR or DWEA. We tried restricting the analysis to those with a length of hospitalisation longer than one day, as a proxy for severity, but this did not change the findings (data not shown).

Age distribution in the study population would not influence the results, as the matching also included age. Misclassification of the outcome cataracts is unlikely, and if present, it would be non‐differential. Another possible limitation is that DNPR includes only hospital contacts, whereas cataract can be treated in outpatient clinics, that do not report to DNPR. However, this underestimation of cataracts is most likely non‐differential and thus not causing bias.

The choice of match persons may also be a limitation, as identifying the optimal match person was challenging. Due to the heterogeneity in severity of electrical injuries, the type of match should reflect the same heterogeneity and at the same time be frequent in all age group to identify a sufficient number of match persons. Our approach was to conduct two different matches: one with another type of injures patients with an injury not related to the eye region and another with population‐based match persons in the same occupation group to reflect same socioeconomic position. The first match may not include patients with a life‐threatening condition such as electrical injury. The second match may cause an overestimation, since they did not have the same care seeking behaviour as the electrical injured persons identified due to their hospital contact.

In 1985, Saffle et al. reviewed the incidence and characteristics of cataracts in 113 burn centre patients with severe electrical injuries (Saffle et al., 1985). Cataracts occurred 1 to 12 months following injury (mean 3.85 months) among 6.2% of the patients. This incidence of cataract was identical to that found by Solem et al., who noted cataracts in 4 of 64 similar patients (7 eyes, 6.3%) (Solem et al., 1977). On the other hand, Skoog reported only 1 patient with cataracts of 141 electrical injury patients treated in the department of reconstructive surgery, equivalent to an incidence of 0.7% with follow‐up over several years (Skoog, 1970). However, 40% of these injuries were from sources of 280 volts or less.

Duman et al. (2015) reported the case of a woman who suffered a low‐voltage injury. When admitted, she went into cardiac arrest, and during four weeks of hospitalisation, the patient gradually developed unilateral uveitis, cataract and retinal detachment. Duman et al. recommended awareness to these rare complications. In particular, it was recommended that patients who had experienced electrical injuries affecting the head and neck should be monitored by an ophthalmologist.

A relatively new cause of electric cataract is following the use of Tasers. Seth et al. reported the case of a 35‐year‐old man who sustained a minor skin burn on the left upper eyelid after being tased and a week after developed a left eye cataract (Seth et al., 2007). Since then, several case reports have described how Tasers may cause eye injury without direct globe trauma (Gapsis et al., 2017; Moysidis et al., 2019).

In a 2013 Canadian clinical review addressing general practitioners, one of the editors key points was, “As many as 6% of those suffering electrical injury will develop cataract in the first year following the exposure, with a smaller number of additional patients developing cataract within 3 years” (Wesner & Hickie, 2013). Also, Reddy's, 1999 electric cataract report stressed the need for cataract screening in all cases of electrical injuries (Reddy, 1999).

Despite these recommendations, this routine cataract screening is not supported by our analysis. It may be that previous case studies and patient group studies do not distinguish sufficiently between electric shocks near the eye and other electrical injuries. The results may be generalised, with the above‐mentioned limitations, to all populations exposed to any electrical injuries.

## 5 CONCLUSION

A review of the literature clearly substantiates the occurrence of electric cataract as a consequence of electric current coming in contact with a point on the skull or near the eye. However, our results indicate that electric cataract is not a delayed‐onset effect of electrical injury, in general, and do not indicate a need for cataract screening in all cases of electrical injury.
